# Effect of Al Content on Synthesis, Mechanical Properties and Corrosion Resistance of Ti_3_(Si,Al)C_2_ MAX Solid Solutions

**DOI:** 10.3390/ma19194057

**Published:** 2026-09-23

**Authors:** Mingdong Bao, Jiahuan Hu, Yingwen Cao

**Affiliations:** School of Materials and Chemical Engineering, Ningbo University of Technology, Ningbo 315211, China

**Keywords:** MAX phase, substitution, mechanical properties, electrochemical behavior

## Abstract

MAX-phase solid solution has attracted much attention for research dealing with complex and harsh working conditions. Solid solutions in a Ti-Al-Si-C system were fabricated from Ti, Si, TiC and Al powders by hot press sintering at 1400 °C for 2 h. Phase identification and micro-morphology were systematically characterized, followed by nano-indentation for mechanical tests and electrochemical tests in NaOH for alkali corrosion tests. The c-lattice parameter increased linearly while the a-parameter was essentially unchanged, which followed Vegard’s law and also suggested strong anisotropy of MAX-phase lattices. The addition of Al element caused a solid-solution-strengthening effect, increasing the nano-indentation and elastic modulus. In the electrochemical tests in NaOH solution, with the increased Al content, the corrosion tendency gradually decreased with the increasing charge transfer obstruction, which suggested improved corrosion resistance.

## 1. Introduction

Ti_3_SiC_2_ is one of the most studied M_n+1_AX_n_ (MAX) phases. The nanolayered ternary compound M_n+1_AX_n_ (MAX) phase is a series of carbides or nitrides with hexagonal crystal structures and a P63/mmc space group, where M refers to the transition metal element, A is the main group element, X is either C or N, and n = 1~3 [1,2,3]. In Ti_3_SiC_2_, C atoms are located at the center of the octahedral Ti-atomic framework, which leads to a stable Ti_6_C structure due to the strong Ti-C covalent bonds. The Si atom (i.e., A-site atom) layers are sandwiched between Ti_6_C layers connected by weaker Ti-Si metal bonds [4,5]. The highly energetic Ti-C covalent bonds give Ti_3_SiC_2_ high hardness and strength [6,7], while the Ti-Si metallic bonds allow Ti_3_SiC_2_ to possess excellent electrical and thermal conductivity [8,9]. Meanwhile, in Ti_3_SiC_2_, both the nanolayered structure and weaker Ti-Si bonds cause graphite-like sliding behavior between layers, which reduces friction resistance and correspondingly contributes to outstanding self-lubrication [10,11]. Thus, Ti_3_SiC_2_ has been applied widely as a multi-functional structural material on many occasions, such as in filters [12], fuel cells [13], nuclear fuel cladding [14] and aerospace turbine components [15].

In order to cope with complex and harsh working conditions, the MAX-phase solid solution formed by elemental substitution on sites has attracted much attention to establish its comprehensive properties [16,17,18]. Arya et al. [19] found that the hardness of V_2_As_1−x_Ge_x_C was significantly higher than V_2_AsC following the incorporation of P. Zhang et al. [20] prepared Ti_2_AlSnC coatings on 316L substrates and demonstrated that Ti_2_AlSnC coatings exhibited more electrical conductivity than Ti_2_AlC due to the atomic vacancy formation energy and the migration energy found by theory calculations. Burr et al. [21] theoretically studied the solubility of Cr in Ti_3_AlC_2_ and found that increased stability of (Cr,Ti)_3_AlC_2_ was detected with the formation of Cr-C bonds, the charge redistribution in the Al layer and the magnetization. Tunca et al. [22] introduced Ti element into a Zr-Al-C system during pressure-less sintering, which reduced the secondary carbides and ultimately enhanced the purity of sintered MAX phases. Hadi et al. [23] found that the substitution of Mo led to lattice deformation caused by the radius differences between Ti and Mo atoms and the simultaneous grain refinement, which enabled Mo_0.8_Ti_0.2_AlC to exhibit more outstanding mechanical properties than Ti_2_AlC. Moreover, the further substitution of multiple elements to obtain the medium- [24,25,26] and high-entropy MAX phase [27,28,29] were also attracted widely to achieve a comprehensive improvements on properties.

Ti_3_(Si,Al)C_2_ is one of the most frequently studied MAX-phase solid solutions, due to the similarity of lattice structures between Ti_3_AlC_2_ and Ti_3_SiC_2_ [30,31,32]. The additional Al substitution during the fabrication of Ti_3_SiC_2_ could inhibit the content of carbides and then improve the purity of target products [33]. Du et al. [34] clarified the effects of Si addition on the wear behavior of Ti_3_AlC_2_ and found that the optimized compound Ti_3_Al_0.6_Si_0.4_C_2_ showed a 25% lower average wear rate than Ti_3_AlC_2_ during the tribo-corrosion tests against GCr15 in nitrate-based molten salts. Du et al. [12] fabricated porous Ti_3_Si(Al)C_2_ by free casting and pressure-less sintering, and found that the oxidation rate of the sample x_Al_ = 0.24 was just 2/5 of that of Ti_3_SiC_2_ after 900 °C oxidation. Hadi et al. [35] studied the continuous changes to structural, electrical and mechanical properties on Ti_3_Al_1−x_Si_x_C_2_ with the varying Si content, and found that both the elastic constants and modii increased with the increasing Si addition. In our previous study [36], porous Ti_3_Al_1−x_Si_x_C_2_ solid solutions were prepared with Ti/Al/Si/TiC powder mixtures by pressure-less sintering. With increasing Si addition, the porosity and pore structure of sintered Ti_3_Al_1−x_Si_x_C_2_ showed a “V”-shaped tendency. Porous Ti_3_Al_0.4_Si_0.6_C_2_ was also employed as the novel wick material for making capillary wicks, and it exhibited excellent heat transfer capacity in loop heat pipes [37]. 

In this work, a Ti_3_(Si,Al)C_2_ solid solution was fabricated by adding varying amounts of Al powder based on the synthesis of Ti_3_SiC_2_ with Ti, Al and TiC powders. Systematic characterizations of the phase identification, lattice parameters and microstructure were carried out. The effects of Al addition on the mechanical properties of Ti_3_(Si,Al)C_2_ solid solutions were also investigated. Both the alkaline corrosion behavior of Ti_3_(Si,Al)C_2_ and effects of Al addition were also explored. The c-lattice parameter increased linearly while the a-parameter was essentially un-changed, which followed Vegard’s law and also suggests the strong anisotropy of MAX phase lattices. The addition of the Al element caused a solid-solution strengthening effect, raising the micro-hardness and elastic modulus. In the electrochemical tests in NaOH solution, with the increased Al content, the corrosion tendency gradually decreased with the increasing charge transfer obstruction, which suggested improved corrosion resistance.

## 2. Experimental Details

Ti_3_(Si,Al)C_2_ MAX solid solutions were fabricated using Ti (~40 μm, 99.9%), Al (~10 μm, 99.9%), Si (~9 μm, 99.9%) and TiC (~ 10 μm, 99.5%) powders as starting materials. In order to offset the evaporation loss of A-site elements (Al and Si) and increase the purity of sintered products, the off stoichiometric molar ratio shown in Table 1 was utilized for powder mixtures. The powder mixtures were mixed by ball milling for 2 h and sintered by hot pressing in vacuum (ZY-40-20Y, Shanghai Chenhua Science Technology Corp., Ltd., Shanghai, China) at 25 MPa at 1400 °C for 2 h. The samples were cut for tests and measurements. The surfaces of samples were all wet-ground with SiC emery paper and then cleaned with acetone using an ultrasonic cleaner before drying.

XRD (X-ray diffraction, Bruker D8 Advance, Karlsruhe, Germany) at 40 kV, 30 mA with Cu Kα radiation was utilized for the phase identifications, with 0.02° per step and a 2θ scan range 5~80°. The lattice parameters of samples were calculated using MDI JADE 6 software. The microstructure and element distribution were observed by scanning electron microscope (SEM, S4800, Hitachi, Tokyo, Japan) with energy dispersive spectroscopy (EDS, Aztec UltimMax 100, Oxford Instruments, Abingdon, UK). The mechanical properties were performed with Nano Indenter G200X (KLA Corporation, Milpitas, CA, USA) at a 40 mN load held for 5 s. Six points were randomly tested for each sample and the average value was taken.

The corrosion resistance was studied with electrochemical tests via a CS350H electrochemical workstation (Wuhan CorrTest Instruments Corp., Ltd., Wuhan, China) at R.T. in the 1 mol/L NaOH aqueous solution. The tree electrode system was used with the saturated calomel electrode as the reference electrode, the platinum mesh electrode as the counter electrode and the Ti_3_Si_1−x_Al_x_C_2_ sample as the working electrode. The open circuit potential (OCP) was monitored as the natural potential of Ti_3_(Si,Al)C_2_ samples in the solutions as a function of time. The potentiodynamic polarization curves were carried out in the scanning range of −1~3.5 V relative to OCP and a rate of 1 mV/s. The electrochemical impedance spectra (EIS) curves were characterized at OCP using a sinusoidal wave with an amplitude of 10 mV in a frequency range from 100 kHz to 0.01 Hz.

## 3. Results and Discussions

Figure 1 shows the XRD results of sintered products. As x_Al_ = 0, i.e., the starting materials were Ti, Si and TiC powders, the products mainly consisted of Ti_3_SiC_2_ and little impurities (tic). Based on the K-value approximation [38], the Ti_3_SiC_2_ content was 88.82%. According to the reaction process of Ti_3_SiC_2_, tic was the intermediate product and partially remained in the sintered samples due to the evaporation loss of Si element in solid–solid synthesis reactions [39,40]. As the Al content increased, the diffraction peaks of TiC gradually weakened, suggesting that the TiC impurity content declined. After the addition of Al element, an Al-Si eutectic with low melting temperature formed [41], thereby bringing the liquid phase into the synthesis process and changing the synthesis reactions from solid–solid reactions to solid–liquid reactions [39]. The liquid phase with good mobility promoted the contact of reactants and correspondingly increased the diffusion of elements, which could thoroughly and comprehensively facilitate the synthesis reactions and subsequently enhance the purity of the MAX phase. In addition, no impurity containing Al element was observed in Figure 1, thereby showing no solution limitation of Al elements in Ti_3_(Si,Al)C_2_. With the Al incorporation, the diffraction peaks gradually and serially varied to a smaller angle. The incorporated Al atoms located at A-sites together with Si atoms, which brought the Ti_3_(Si,Al)C_2_, substitutional solid solutions. The layer spacing of the MAX phase was enlarged due to Al incorporation, since Al atoms (143 pm) are larger than Si atoms (117 pm). This brought about shifts in diffraction peaks of the MAX phase, and also the formation of continuous Ti_3_(Si,Al)C_2_ solid solution and the conversion from Ti_3_SiC_2_ to Ti_3_AlC_2_.

Based on the XRD patterns above, the lattice parameters were calculated by MDI JADE software. The lattice parameters of Ti_3_SiC_2_ in this work were a = 0.30631 nm and c = 1.73511 nm, which agreed with the PDF card of Ti_3_SiC_2_ (No. 48-1826): a = 0.30654 nm, c = 1.7665 nm. So did Ti_3_AlC_2_; the lattice parameters of Ti_3_AlC_2_ in this work were a = 0.30774 nm, c = 1.85679 nm, which agreed with the PDF card of Ti_3_AlC_2_ (No. 52-0875): a = 0.3069 nm, c = 1.8501 nm. In Figure 2, as the addition of Al increased, the a-lattice parameter saw little change while the c-lattice parameter gradually increased. The radius of Al atoms (143 pm) was bigger than that of Si atoms (117 pm), thereby increasing the layer spacings between MX layers and A-site layers along the c-axis and finally enlarging the c-lattice parameter. Moreover, the lattice parameters showed a linear relationship with the addition of Al element, which demonstrated the formation of solid solutions according to Vegard’s law (the lattice parameters of the substitutional solid solutions are linearly dependent on the molar fractions of its components) [42,43]. With the increased Al content, the lattice parameter ratio c/a shown in Figure 3 gradually increased. This suggested that after the substitution of Al atoms, the lattice elongation along the c-axis was relatively larger than that along the a-axis, thereby demonstrating the high anisotropy of the MAX phase.

Typical morphologies of solid solutions are shown in Figure 4. Both the solid–solid synthesis reactions of Ti_3_SiC_2_ and the unavoidable gaps between powders decreased the density of sintered products and caused the holes which can be seen in Figure 4a. After magnification of the rectangle, the layered structure could be observed in Figure 4b, which could inhibit the crack extension and then enhance the damage tolerance of MAX phases [44]. Figure 5, Figure 6, Figure 7, Figure 8, Figure 9 and Figure 10 show the EDS results of Ti_3_(Si,Al)C_2_ solid solutions. In general, each sample exhibited uniform element distribution. The ratio between M-site atoms (i.e., Ti) and A-site atoms (Al + Si in this work) was coincident with 3:1, and the ratio of Si/Al agreed with the nominal formula Ti_3_Si_1−x_Al_x_C_2_, which indicated that Ti_3_(Si,Al)C_2_ solid solutions could be prepared by sintering Ti, Si, Al and TiC powder mixtures.

Nano-indentation was employed to evaluate the mechanical properties of Ti_3_(Si,Al)C_2_ solid solutions. Figure 11a shows the load-depth curves during the loading and unloading process. During the loading process, elastic deformation occurred at the initial stage; then, as the load increased, the samples underwent plastic deformation. During the unloading process, only elastic deformation was restored, while plastic deformation was not seen, but rather permanent deformation due to the irreversibility. The calculated nano-indentation hardness and elastic modulus of samples are shown in Figure 11b. The hardness and elastic modulus of Ti_3_SiC_2_ were 12.8 GPa and 220.3 GPa, respectively. With the increasing Al incorporation, the nano-indentation hardness of Ti_3_(Si,Al)C_2_ increased first and then decreased, thereby reaching the maximum of 17.4 GPa at x_Al_ = 0.4 (with the nominal molecular formula Ti_3_Si_0.6_Al_0.4_C_2_). The trend of elastic modulus was the same. The elastic modulus reached the maximum of 255.9 GPa at x_Al_ = 0.4 (with the nominal molecular formula Ti_3_Si_0.6_Al_0.4_C_2_), which was higher than two ends (220.3 GPa for Ti_3_SiC_2_ and 237.5 GPa for Ti_3_AlC_2_). After the substitution, Al and Si atoms were simultaneously located at the A-site layers. The atomic radius difference between Al and Si atoms contributed to the lattice expansion, enlarged the difficulty of dislocation glide, and correspondingly drove the solid solution hardening effect [45,46,47], which led to higher nano-indentation hardness of Ti_3_(Si,Al)C_2_ compared to the two ends.

The Al elements alloyed into Ti_3_SiC_2_ occupied A-site layers together with Si, correspondingly forming a substitutional solid solution. Since the atomic radius of Al (143 pm) was larger than that of Si (117 pm), the substitution enlarged the spacings between the MX and A-site layers, which led to lattice expansion and enhanced the dislocation motion resistance. This contributed to the solid-solution strengthening effects and made the hardness of the solid solution higher than that of its end members. This solid-solution strengthening effect brought about by elemental incorporation in the MAX-phase solid solutions was also found in Refs. [19,23].

Electrochemical corrosion occurred when the materials came into contact with an electrolyte solution due to the formation of a galvanic cell. The three-electrode system is the most commonly used testing system in electrochemical testing to evaluate the corrosion performance of materials. In this work, the MAX-phase solid solutions acted as the working electrode, the platinum mesh electrode acted as the counter electrode and the saturated calomel electrode was the reference electrode. To characterize the changes in samples when contacted with solution, open circuit potential (OCP) curves were employed by testing the real-time natural potential of samples in the solution [48]. A positive shift indicated the formation of a protective passive film which inhibited the corrosion reactions on anodes and correspondingly enhanced the corrosion resistance. In contrast, a negative shift suggested that the passive film was damaged or dissolved partially, thereby making anodic reactions easier, increasing the corrosion tendency and reducing the corrosion resistance. Figure 12 shows the OCP curves of Ti_3_(Si,Al)C_2_ samples in NaOH solution. In Figure 12a, the OCP of Ti_3_SiC_2_ consisted of two stages: (1) firstly, because of exposition to air, an unstable oxide layer formed on Ti_3_SiC_2_ and then dissolved upon contact with the NaOH solution, thereby reducing the potential [49]; (2) at 160 s, the OCP recovered since a new passive film formed on the freshly exposed Ti_3_SiC_2_ surface, which reduced the activity of Ti_3_SiC_2_ surface and then raised the potential. Although the OCP of Ti_3_SiC_2_ exhibited a “V”-shaped variation, it remained essentially stable with a small overall difference (only 0.015 V). In Figure 12b, with increased substitution of Al, the duration of stage 1 shortened. When x_Al_ = 1.0, i.e., the sample Ti_3_AlC_2_, the initial drop in stage 1 disappeared. Meanwhile, the recovery of OCP increased progressively with the increasing substitution of Al. This could be explained by the formation of a more effective passive film on the surface and its correspondingly strong protective effects.

Polarization curves, gained from the dynamic potential scanning on the working electrode, were used to evaluate the corrosion performance and passive behaviors of materials. Potential-dynamic polarization curves in Figure 13 were carried out to systemically study the corrosion behavior of Ti_3_(Si,Al)C_2_. The curves in the anode region consisted of three stages: taking Sample A-0.4 as Example (1) in activation zone (−0.784~0.217 V), the current density rose up with the increment of potential, which suggested that the active dissolution occurred on the surface; (2) in the active-passive zone (−0.217~0.022 V), the current grew slowly and an incomplete oxide film started to form on the surface; (3) in the passive zone (>0.022 V), the current density essentially remained constant with the formation of stable oxide film on the surface, which effectively protected the samples from the corrosion solution. The passive platform has also been observed in other studies which focused on the corrosion behaviors of Ti_3_SiC_2_ and Ti_3_AlC_2_ in NaOH solution [49,50].

Tafel fitting was employed to further analyze the polarization curves. Table 2 lists the calculated self-corrosion potential and self-corrosion current density *I_corr_* of Ti_3_(Si,Al)C_2_ samples. The self-corrosion potential *E_corr_* referred to the corrosion tendency, and the higher *E_corr_*, the lower the corrosion tendency. When the substitution of Al increased, *E_corr_* generally reduced, suggesting decreasing corrosion tendency. Meanwhile, the reduction in *I_corr_* suggested that the anodic reactions on Ti_3_(Si,Al)C_2_ samples weakened, subsequently indicating the increment of corrosion resistance, i.e., the A-site substitution of Al could improve the alkaline corrosion resistance of Ti_3_SiC_2_. Moreover, the passive platform in curves (i.e., the passive current density) weakened with increasing Al incorporation, thereby showing the increasing protection of passive films.

Electrochemical impedance spectroscopy is often utilized to analyze the effects of the solution resistance, charge transfer process, passive films and diffusion process on corrosion reactions. Figure 14 shows the impedance results of Ti_3_(Si,Al)C_2_ in NaOH solution. In Figure 14a, the curves at high frequency showed a semi-circle with increasing Al incorporation, and the arc radius of the semi-circle curves at high frequency gradually increased, together with the increment of slopes in impedance curves, suggesting that the inner diffusion resistance of ions increased and the diffusion rate correspondingly increased. Meanwhile, a 45° inclined line appeared in the low-frequency region and represents the Warburg impedance. This indicates that the corrosion products accumulated on the surface of samples, and their diffusion process contributed to the system impedance.

The impedance curves were fitted using the circuit shown in Figure 15. Here, R_s_ represented the resistance of the NaOH solution; Q_f_ and R_f_ were the resistance and capacitance of the surface passivation film generated by the reactions between the sample and NaOH solution, respectively; Q_dl_ was the double-layer capacitance; W was the Warburg impedance, resulting from ion diffusion within the passivation film; and R_ct_ was the charge transfer resistance, where the larger R_ct_, the better the corrosion resistance of the material. Table 3 lists the fitting results of the impedance spectra. As Al incorporation increased, R_ct_ gradually increased, indicating that the resistance of charge transfer between the passivation film and Ti_3_(Si,Al)C_2_ gradually increased, thereby enhancing the corrosion resistance of Ti_3_(Si,Al)C_2_.

According to Refs. [50,51,52], the corrosion mechanism of MAX phases was separated into two stages: (1) firstly, the corrosion medium preferentially attacked the comparatively weaker M-A bonds and invaded the crystals along the channel between M and A layers. This induced the selective dissolution of the A-site atoms and then the corresponding oxides formed. Conversely, the strong covalent M-X bonds contributed to the relatively higher corrosion resistance of M-X frameworks and maintained their integrity; (2) with the further invasion of the corrosion medium, the M-X frameworks dissolved and the oxides of M-site atoms were generated. Whether the A-site oxides were stable or soluble determined the protection of passive films and also the corrosion resistance of MAX phases. Due to the compact and protective Al_2_O_3_ layers, the corrosion resistance of the Al-contained MAX phases were excellent compared to the Si-contained MAX phases [53]. The same trends were observed in this work.

## 4. Conclusions

Ti_3_(Si,Al)C_2_ solid solutions were successfully prepared by introducing varying amounts of Al into the Ti_3_SiC_2_ matrix. After Al incorporation, the a-lattice constant remained basically unchanged, while the c-constant increased linearly. The element distribution in all samples was uniform along with the proportion between elements coincident with the nominal chemical formula as designed. The mechanical tests showed that the atomic difference between Al and Si elements induced the lattice distortion of the MAX phase, resulting in the effect of solid solution hardening. The nano-indentation and elastic modulus first increased and then decreased with the increasing Al incorporation, reaching the maximum at x_Al_ = 0.4 (with the nominal molecular formula Ti_3_Si_0.6_Al_0.4_C_2_), which was higher than the end components. In the electrochemical tests in NaOH solution, as the Al incorporation increased, the self-corrosion potential shifted positively, thereby indicating a reduction in the corrosion tendency. The Ti_3_(Si,Al)C_2_ demonstrated a passive behavior in NaOH solution. The self-corrosion current density decreased with the increasing Al incorporation, and the charge transfer resistance increased, which suggested an enhancement in the charge transfer resistance and the alkaline corrosion resistance.

## Figures and Tables

**Figure 1:**
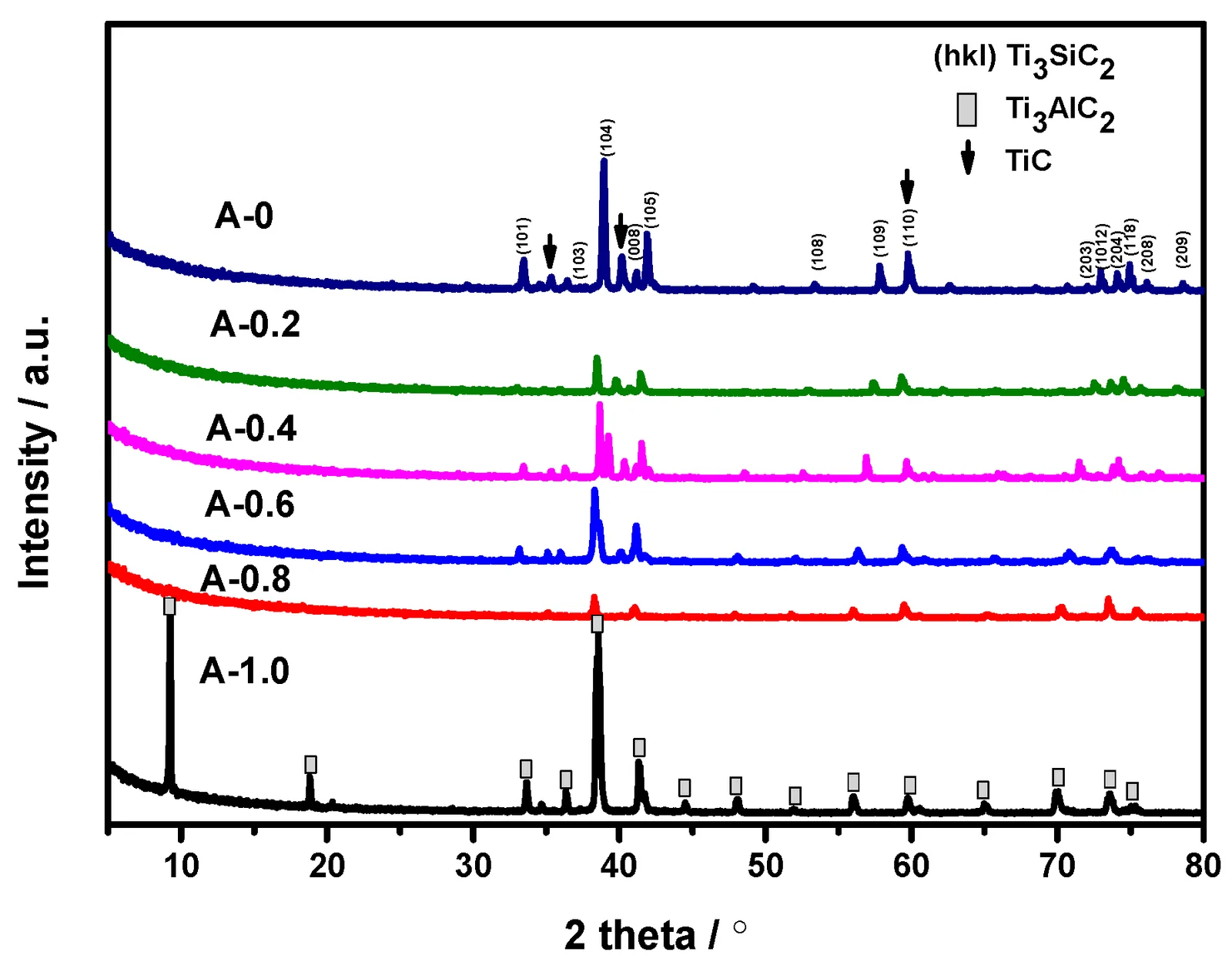
XRD patterns of sintered products.

**Figure 2:**
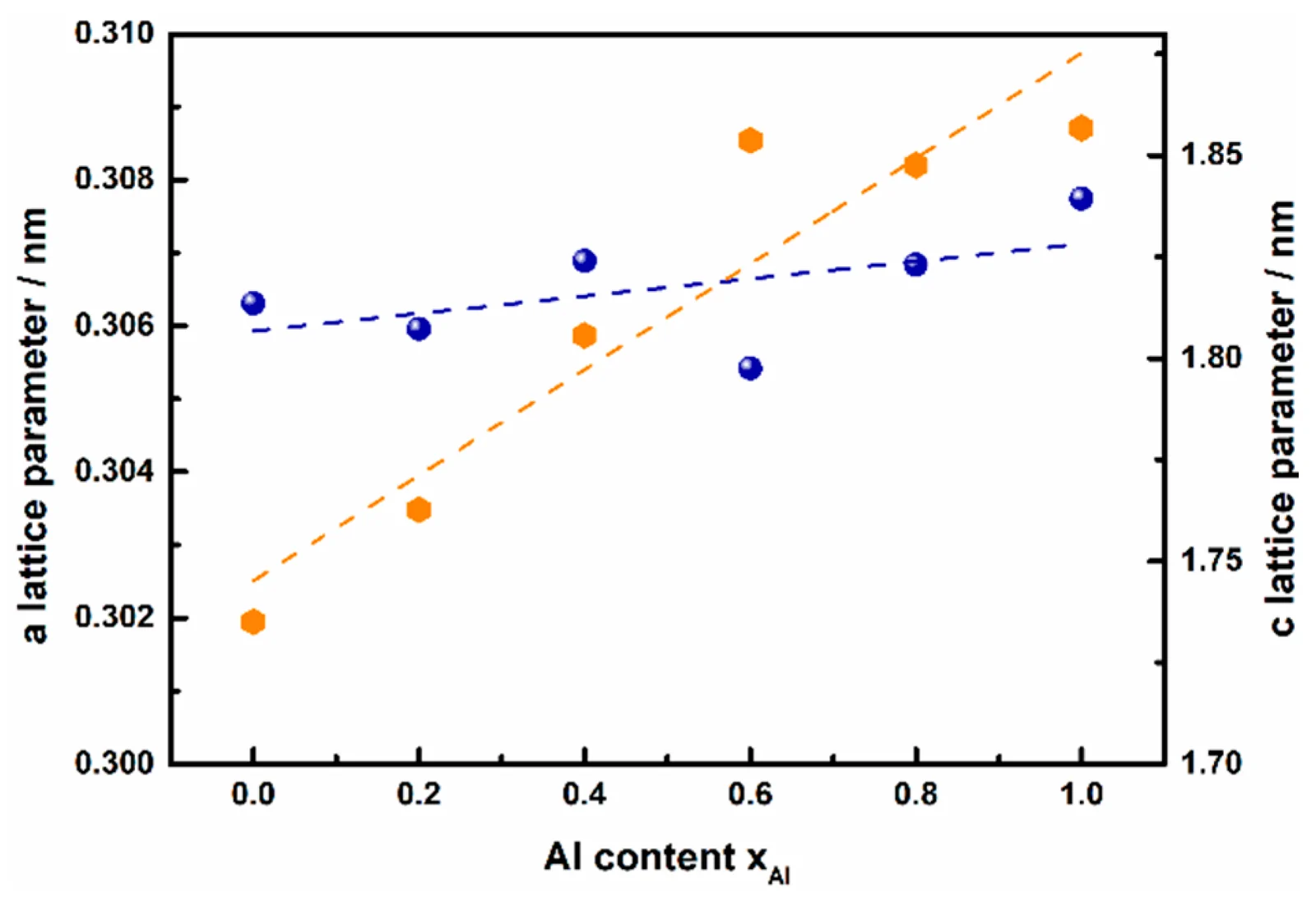
Lattice parameters of Ti_3_(Si,Al)C_2_ solid solutions (with a-parameter shown in blue and c shown in orange).

**Figure 3:**
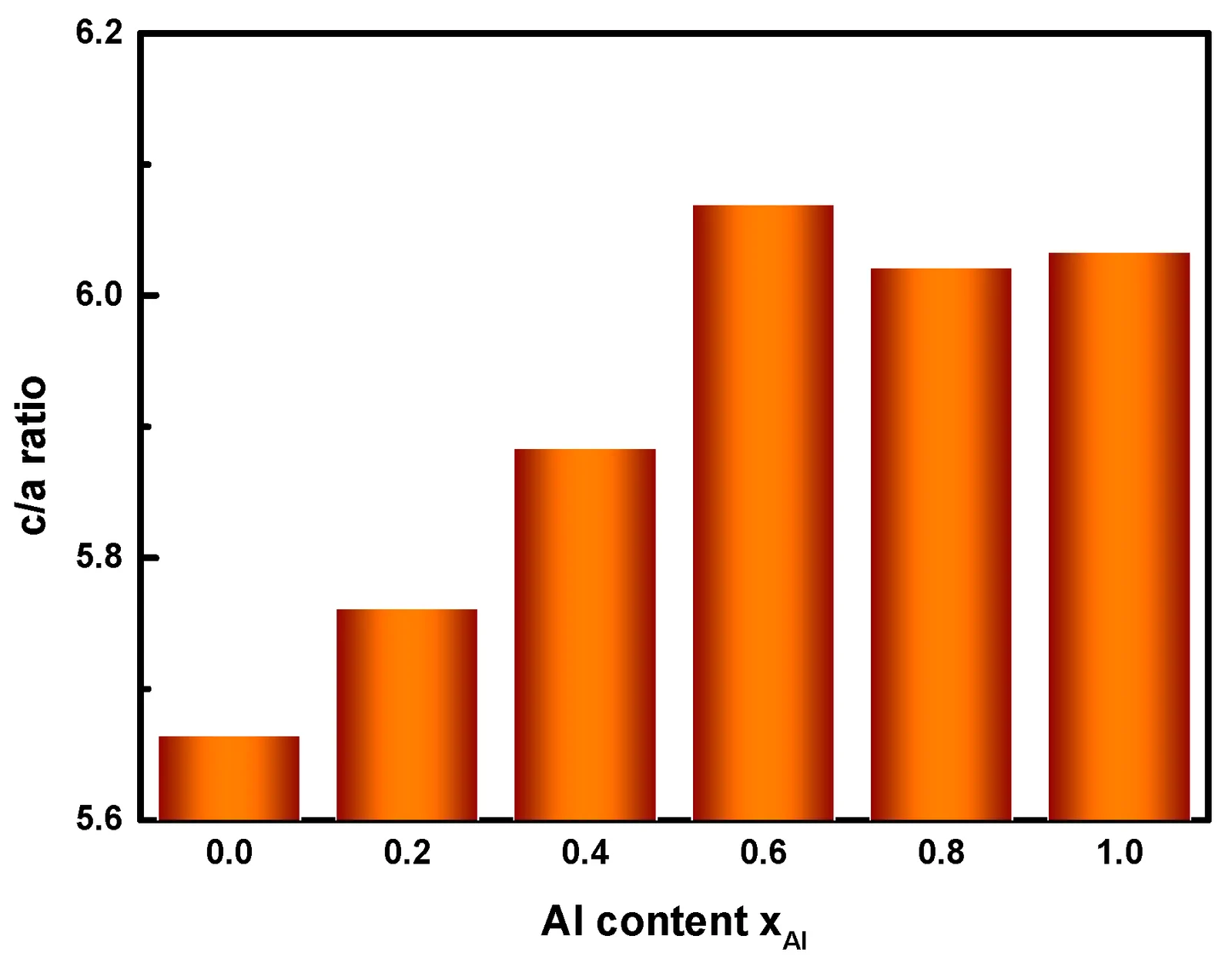
Lattice parameter ratio c/a of Ti_3_(Si,Al)C_2_ solid solutions.

**Figure 4:**
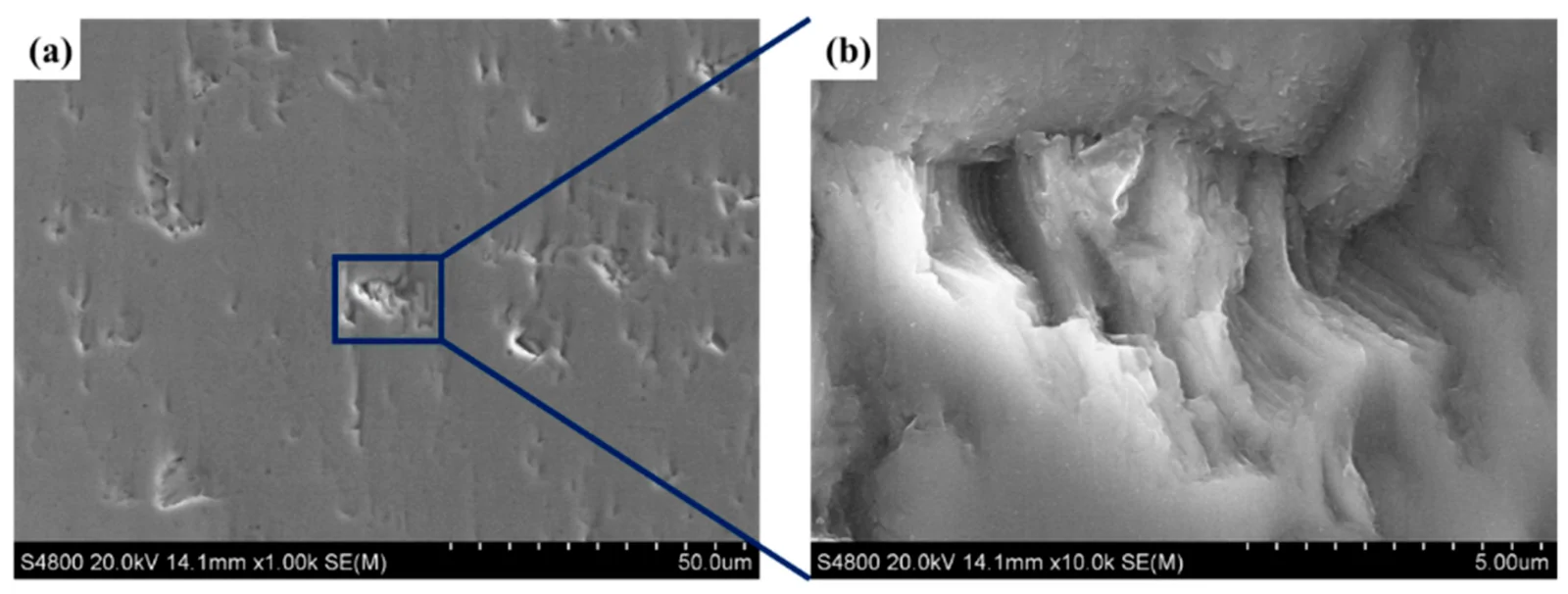
(**a**) Typical morphologies of sintered samples (take sample A-0.2 as an example); (**b**) a magnification of the blue rectangle in (**a**).

**Figure 5:**
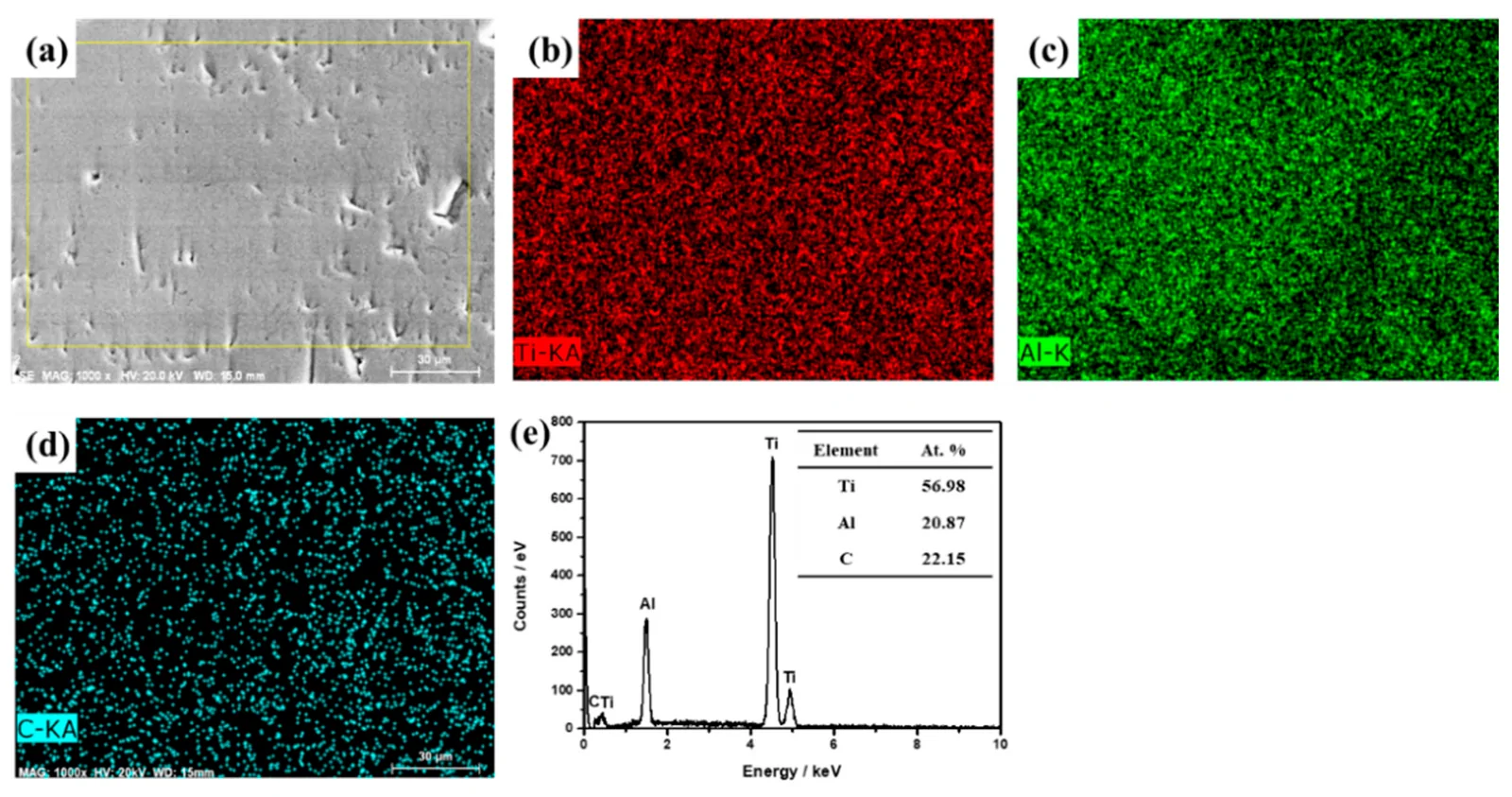
(**a**) Morphology of sintered sample A-0 and (**b**–**e**) EDS results based on the yellow rectangle in (**a**).

**Figure 6:**
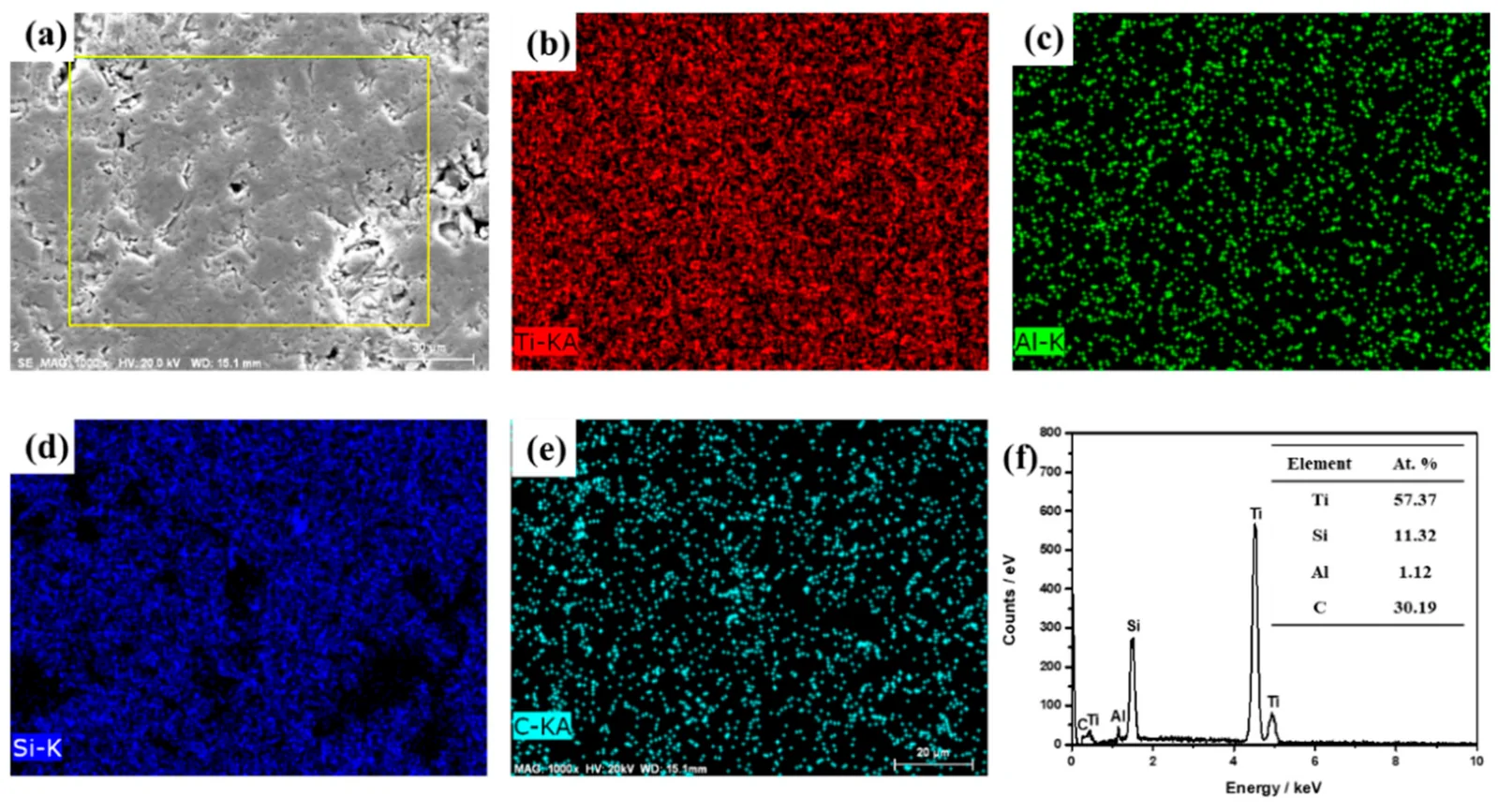
(**a**) Morphology of sintered sample A-0.2 and (**b**–**f**) EDS results based on the yellow rectangle in (**a**).

**Figure 7:**
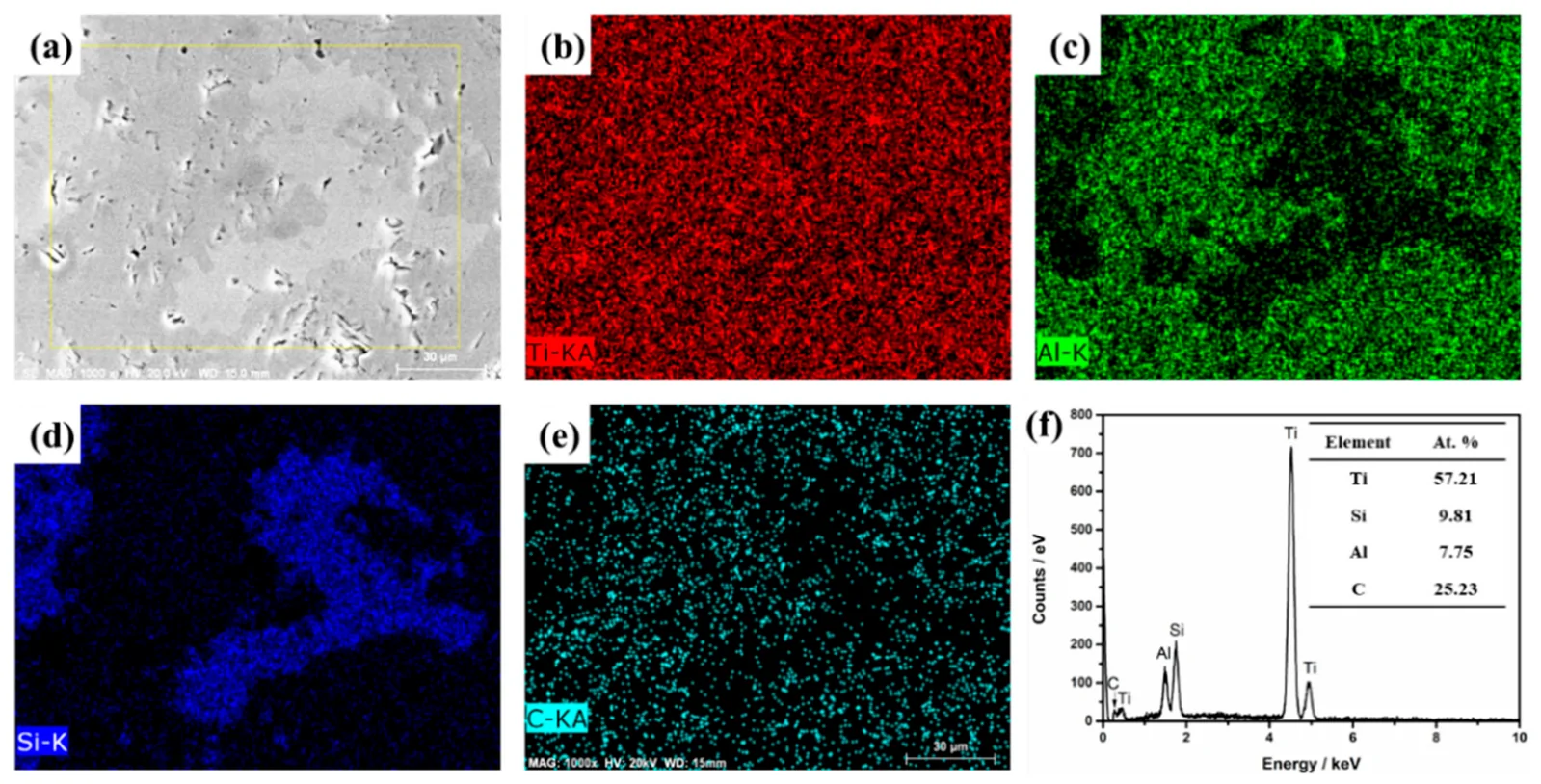
(**a**) Morphology of sintered sample A-0.4 and (**b**–**f**) EDS results based on the yellow rectangle in (**a**).

**Figure 8:**
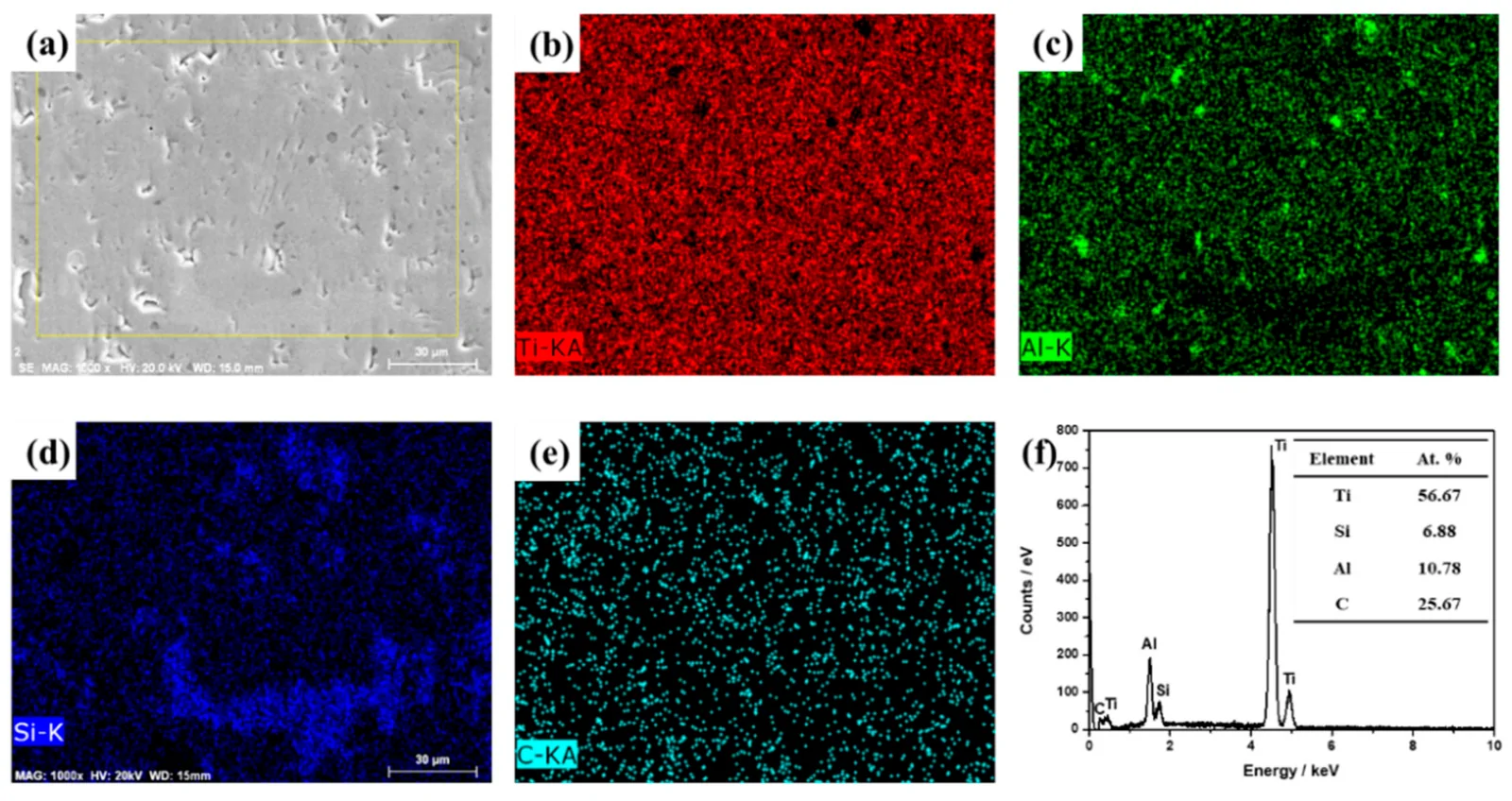
(**a**) Morphology of sintered sample A-0.6 and (**b**–**f**) EDS results based on the yellow rectangle in (**a**).

**Figure 9:**
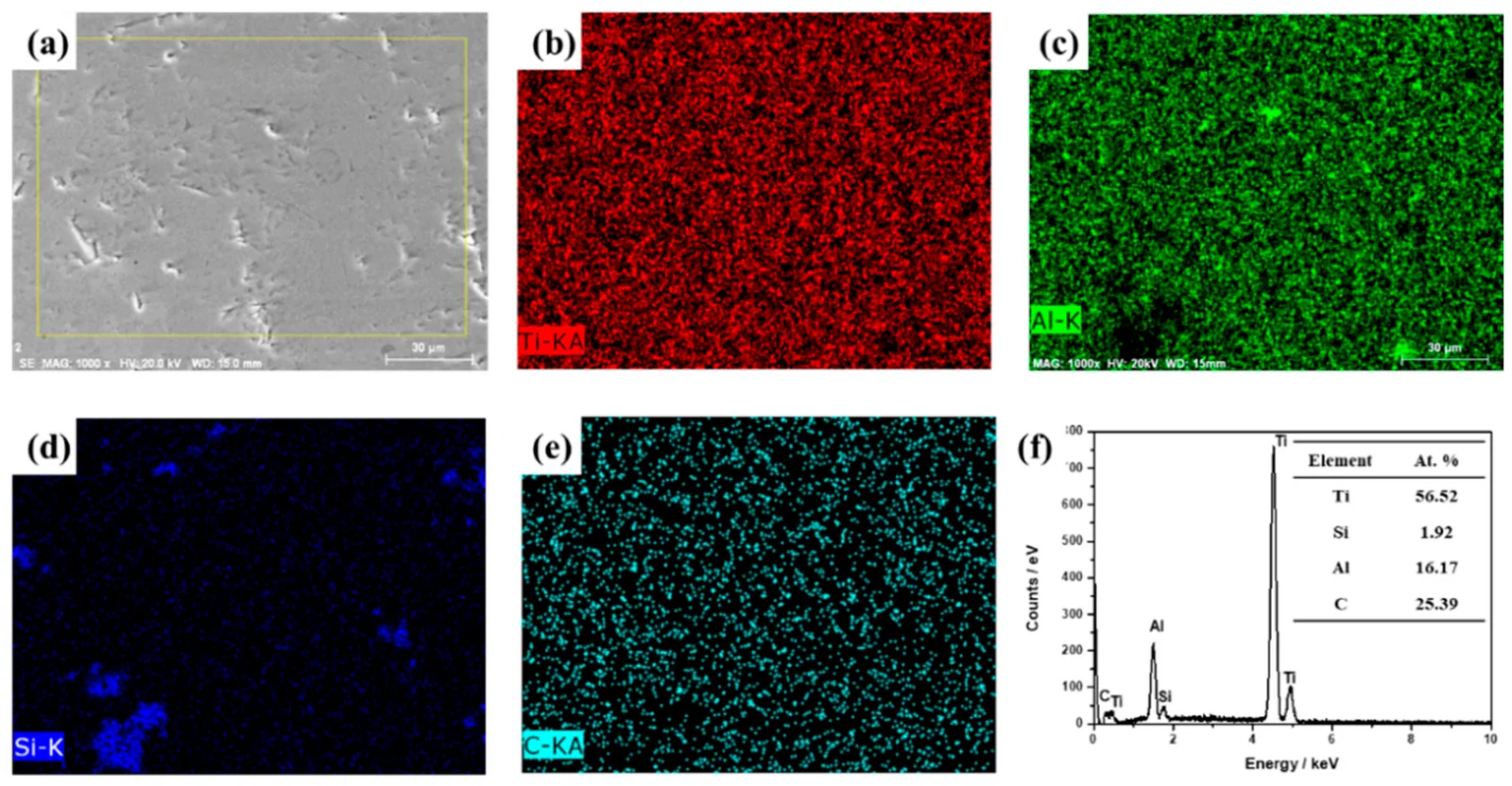
(**a**) Morphology of sintered sample A-0.8 and (**b**–**f**) EDS results based on the yellow rectangle in (**a**).

**Figure 10:**
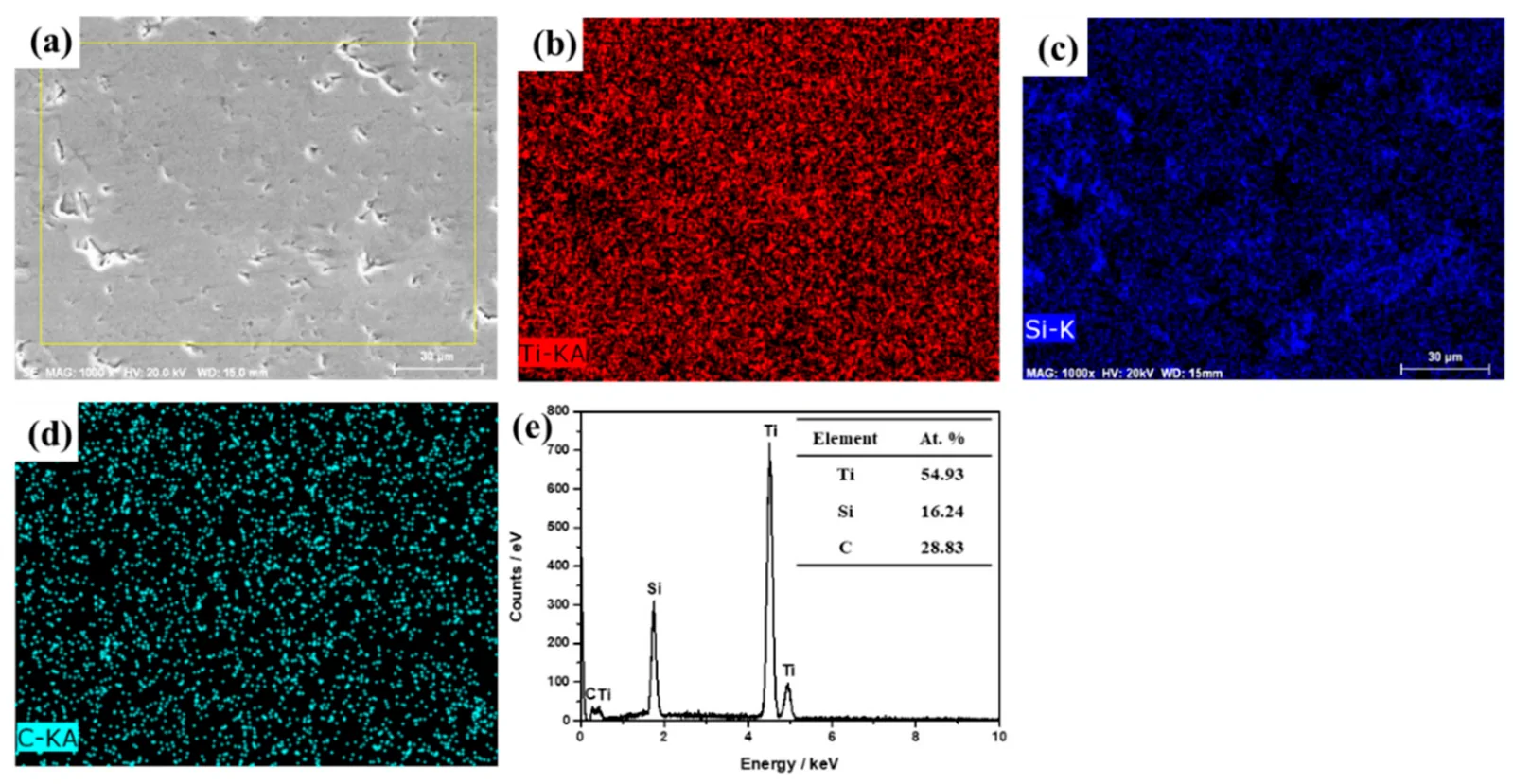
(**a**) Morphology of sintered sample A-1.0 and (**b**–**e**) EDS results based on the yellow rectangle in (**a**).

**Figure 11:**
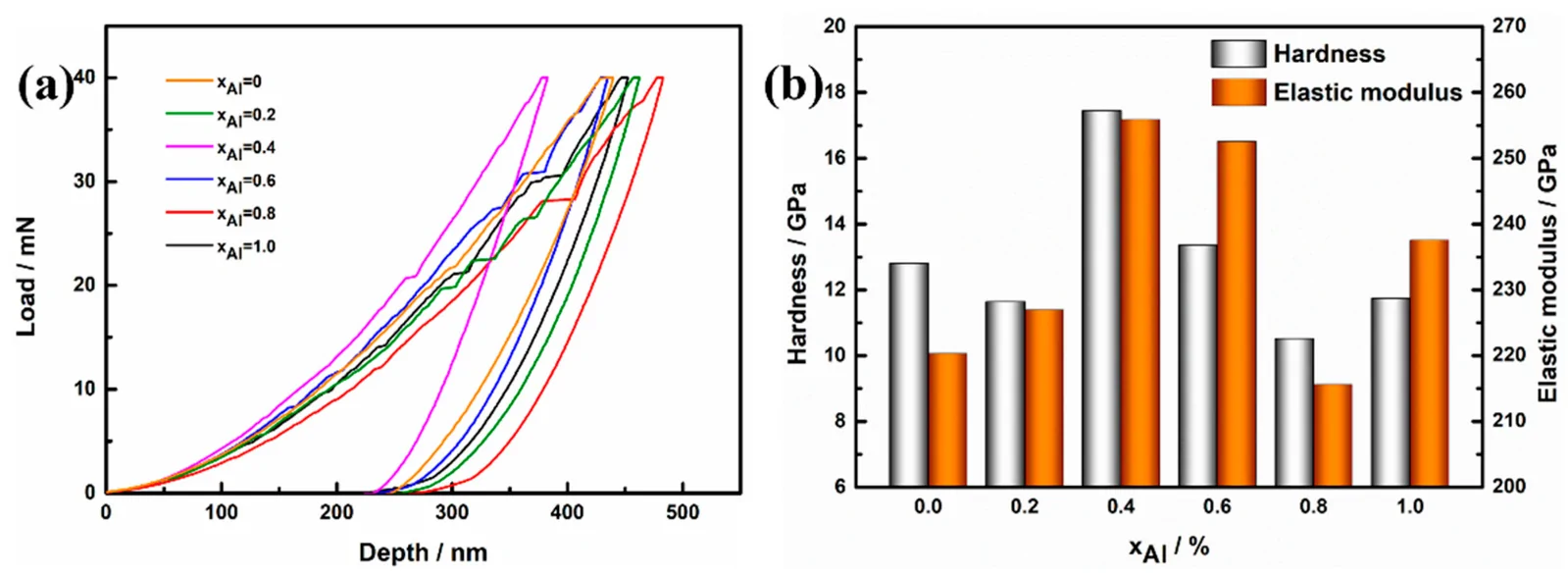
(**a**) Nano-indentation curves and (**b**) calculations of Ti_3_(Si,Al)C_2_ samples.

**Figure 12:**
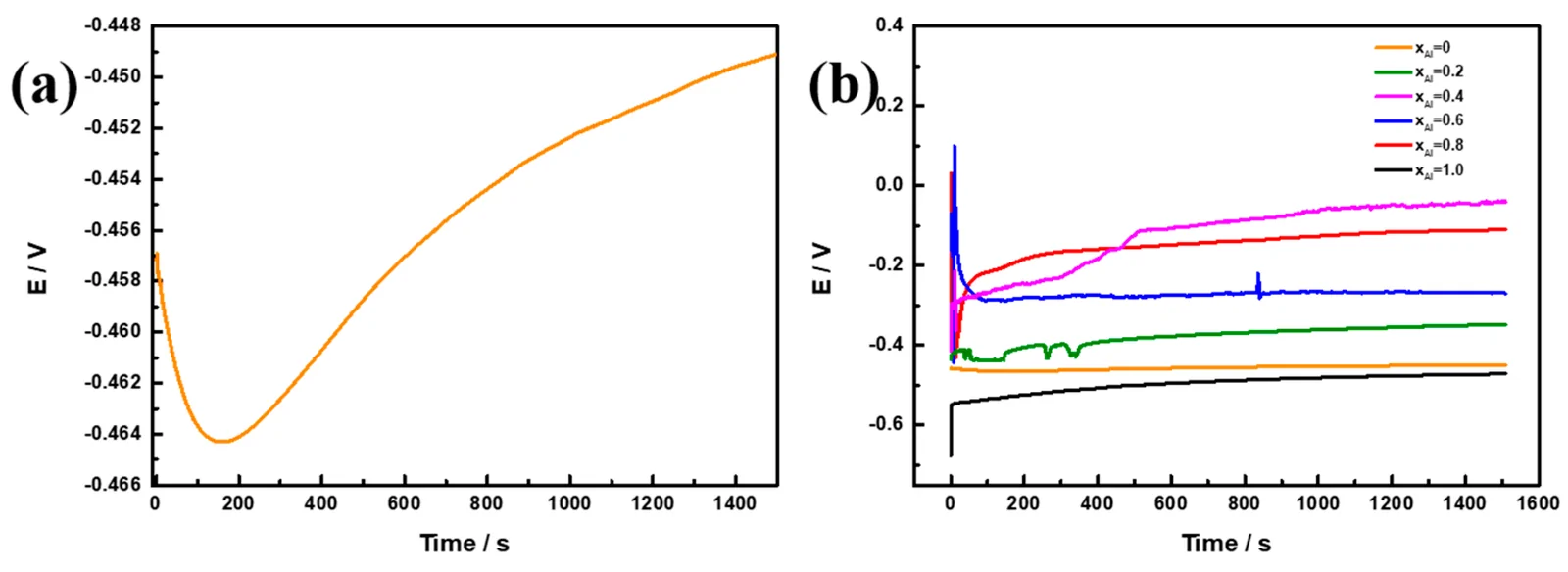
OCP curves of (**a**) Ti_3_SiC_2_ and (**b**) Ti_3_(Si,Al)C_2_.

**Figure 13:**
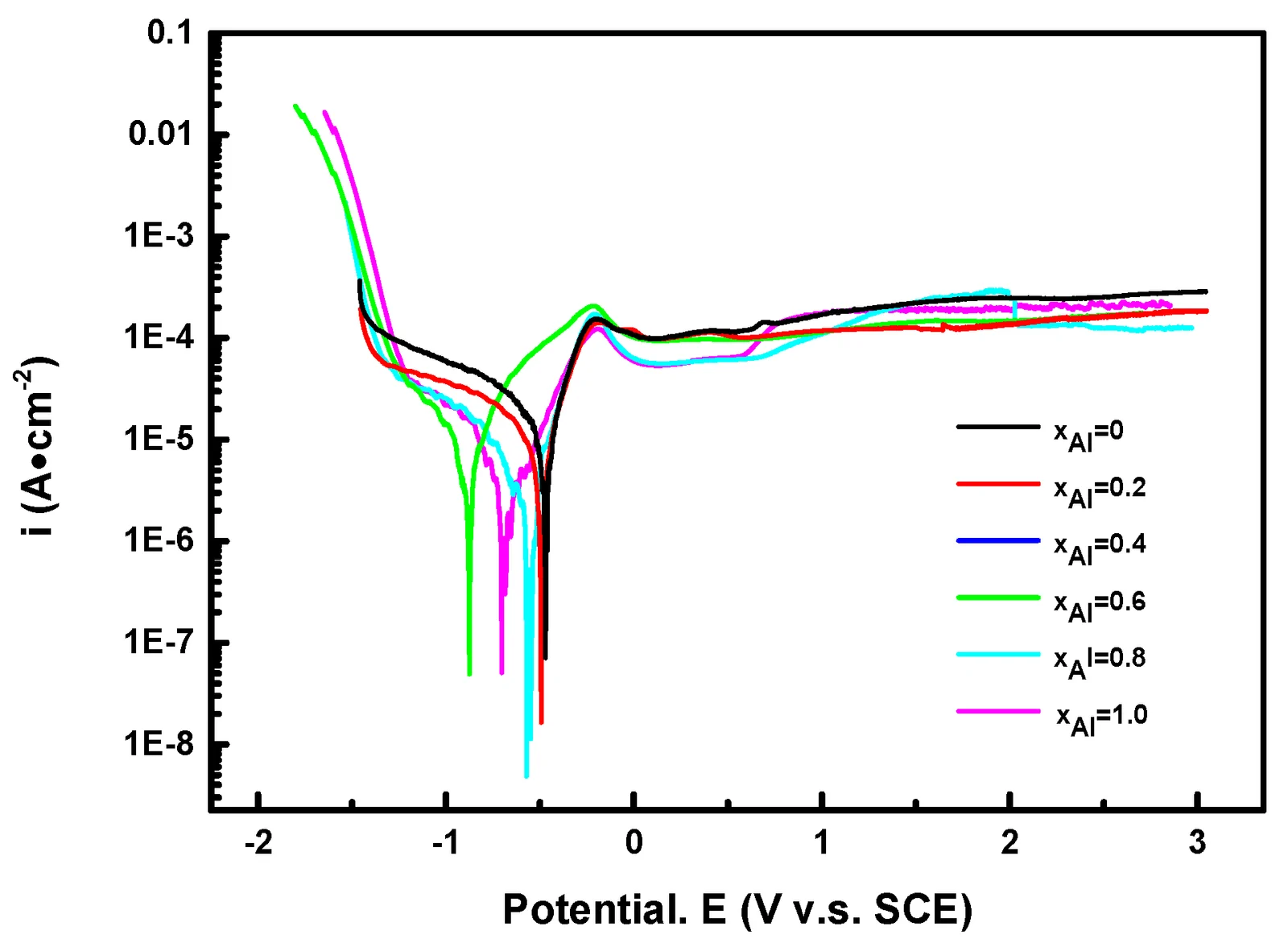
Curves of Ti_3_(Si,Al)C_2_ samples in NaOH solution.

**Figure 14:**
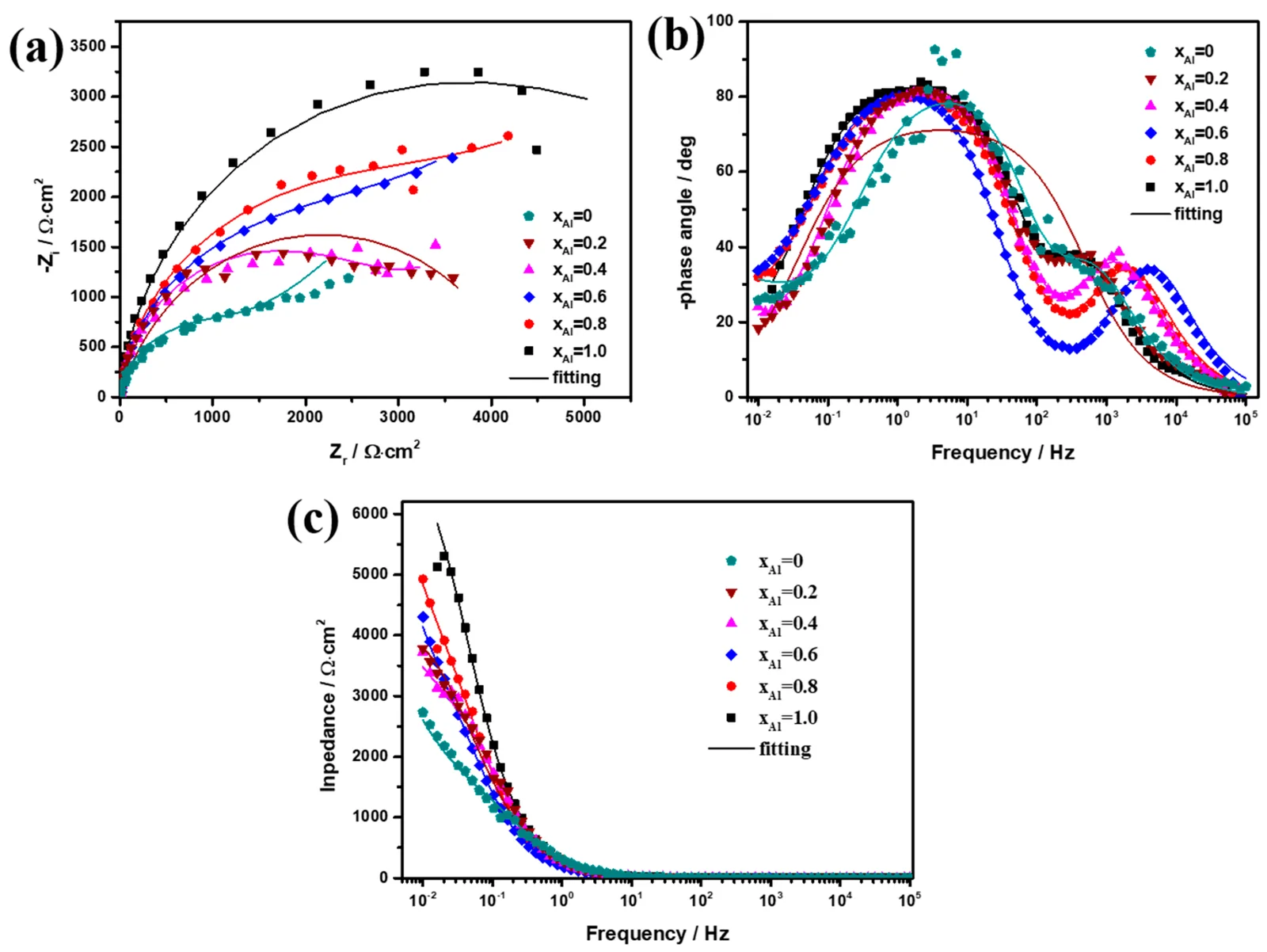
(**a**) Nyquist and (**b**,**c**) Bode plots for Ti_3_(Si,Al)C_2_ in NaOH solution.

**Figure 15:**
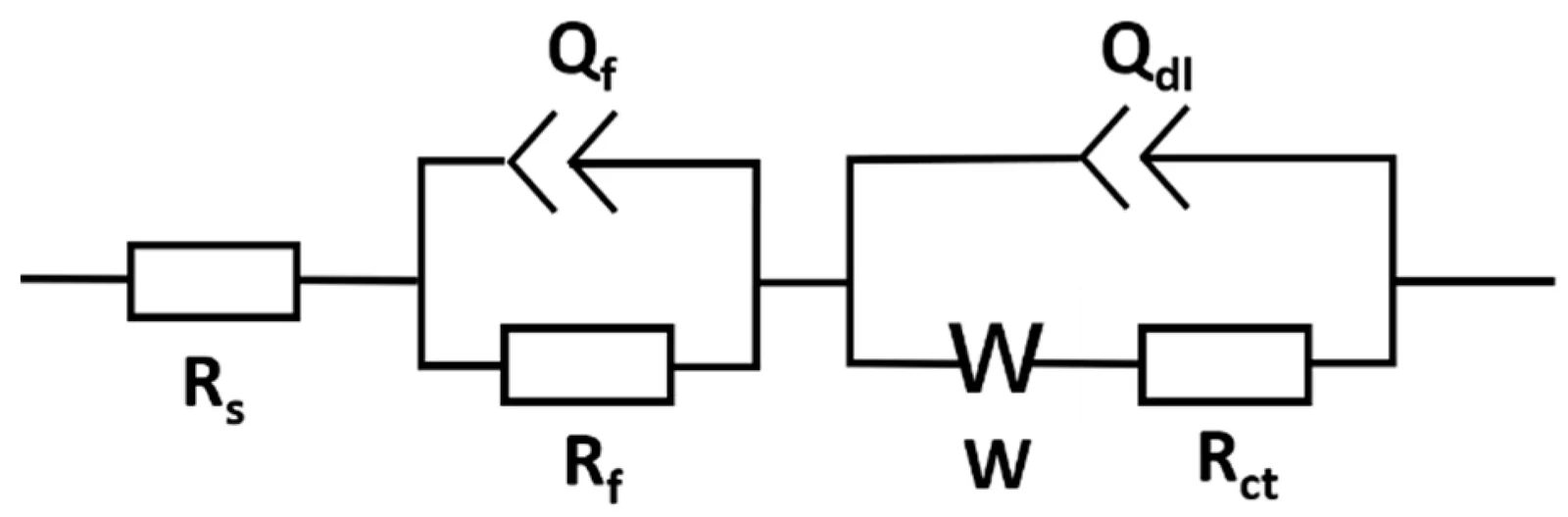
Equivalent circuit for impedance fitting.

**Table 1:** The off stoichiometric molar ratio of powder mixtures.

Sample No.	Molar Ratio (Ti:Al:Si:TiC)
A-0	2:0:2:3
A-0.2	2:0.4:1.6:3
A-0.4	2:0.8:1.2:3
A-0.6	2:1.2:0.8:3
A-0.8	2:1.6:0.4:3
A-1.0	2:2:0:3

**Table 2:** Tafel fitting results of polarization curves in Figure 13.

Sample	Ecorr (V vs. SCE)	Icorr (A·cm^−2^)
A-0	−0.701	1.86 × 10^−6^
A-0.2	−0.742	7.07 × 10^−6^
A-0.4	−0.875	8.25 × 10^−6^
A-0.6	−0.565	8.41 × 10^−6^
A-0.8	−0.493	1.05 × 10^−5^
A-1.0	−0.469	1.82 × 10^−5^

**Table 3:** Fitting results of impedance spectra for Ti_3_(Si,Al)C_2_ samples.

Samples		A-0	A-0.2	A-0.4	A-0.6	A-0.8	A-1.0
Rs (Ω·cm^2^)		1.613	1.922	1.364	1.816	1.505	1.694
Qf	Y (Ω^−1^ s^n^cm^−2^)	3.11 × 10^−4^	7.57 × 10^−4^	1.48 × 10^−4^	2.84 × 10^−5^	8.31 × 10^−5^	3.07 × 10^−4^
	nf	0.8595	0.8154	0.8673	0.9207	0.8973	0.8716
Rf (Ω·cm^2^)		4.106	4.357	6.048	6.121	5.231	7.789
Qdl	Y (Ω^−1^ s^n^cm^−2^)	2.41 × 10^−4^	4.54 × 10^−4^	4.88 × 10^−4^	8.85 × 10^−4^	6.64 × 10^−4^	3.15 × 10^−4^
	ndl	1	0.942	0.9839	0.9578	0.9474	1
Rct (Ω·cm^2^)		1267	2029	2742	2798	3631	6343
W (S-sec^^^5cm^−2^)		2.29 × 10^−3^	1.61 × 10^−5^	3.31 × 10^−3^	1.66 × 10^−3^	1.67 × 10^−3^	2.92 × 10^−3^

## Data Availability

The original contributions presented in this study are included in the article. Further inquiries can be directed to the corresponding author.
